# Neoadjuvant Chemoradiotherapy Followed by Surgery Versus Surgery Alone for Locally Advanced Squamous Cell Carcinoma of the Esophagus (NEOCRTEC5010): A Phase III Multicenter, Randomized, Open-Label Clinical Trial

**DOI:** 10.1200/JCO.2018.79.1483

**Published:** 2018-08-08

**Authors:** Hong Yang, Hui Liu, Yuping Chen, Chengchu Zhu, Wentao Fang, Zhentao Yu, Weimin Mao, Jiaqing Xiang, Yongtao Han, Zhijian Chen, Haihua Yang, Jiaming Wang, Qingsong Pang, Xiao Zheng, Huanjun Yang, Tao Li, Florian Lordick, Xavier Benoit D’Journo, Robert J. Cerfolio, Robert J. Korst, Nuria M. Novoa, Scott J. Swanson, Alessandro Brunelli, Mahmoud Ismail, Hiran C. Fernando, Xu Zhang, Qun Li, Geng Wang, Baofu Chen, Teng Mao, Min Kong, Xufeng Guo, Ting Lin, Mengzhong Liu, Jianhua Fu, on behalf of the AME Thoracic Surgery Collaborative Group

**Affiliations:** Hong Yang, Hui Liu, Xu Zhang, Qun Li, Ting Lin, Mengzhong Liu, and Jianhua Fu, Sun Yat-sen University Cancer Center, Guangzhou; Yuping Chen, Zhijian Chen, and Geng Wang, Cancer Hospital of Shantou University Medical College, Shantou; Zhijian Chen, The University of Hong Kong-Shenzhen Hospital, Shenzhen, Guangdong Province; Chengchu Zhu, Haihua Yang, Baofu Chen, and Min Kong, Taizhou Hospital, Wenzhou Medical University, Linhai; Weimin Mao and Xiao Zheng, Zhejiang Cancer Hospital, Hangzhou, Zhejiang Province; Wentao Fang, Jiaming Wang, Teng Mao, and Xufeng Guo, Shanghai Chest Hospital, Shanghai Jiaotong University; Jiaqing Xiang and Huanjun Yang, Fudan University Shanghai Cancer Center, Shanghai; Zhentao Yu and Qingsong Pang, Tianjin Medical University Cancer Hospital, Tianjin; Yongtao Han and Tao Li, Sichuan Cancer Hospital, Chengdu, Sichuan Province, China; Florian Lordick, University Cancer Center Leipzig, University Medicine Leipzig, Leipzig; Mahmoud Ismail, Academic Hospital of the Charité – Universitätsmedizin, Humboldt University Berlin, Berlin, Germany; Xavier Benoit D’Journo, Aix-Marseille University, Hôpital Nord, Chemin des Bourrely, Marseille Cedex, France; Robert J. Cerfolio, New York University Langone Health; Robert J. Korst, Icahn School of Medicine, Mount Sinai Health System, New York, NY; Robert J. Korst, Valley/Mount Sinai Comprehensive Cancer Care, Paramus, NJ; Nuria M. Novoa, University Hospital of Salamanca, Paseo de San Vicente, Salamanca, Spain; Scott J. Swanson, Brigham and Women's Hospital, Dana-Farber Cancer Institute, Harvard Medical School, Boston, MA; Alessandro Brunelli, St James's University Hospital, Leeds, United Kingdom; and Hiran C. Fernando, Inova Fairfax Medical Center, Inova Schar Cancer Institute, Falls Church, VA.

## Abstract

**Purpose:**

The efficacy of neoadjuvant chemoradiotherapy (NCRT) plus surgery for locally advanced esophageal squamous cell carcinoma (ESCC) remains controversial. In this trial, we compared the survival and safety of NCRT plus surgery with surgery alone in patients with locally advanced ESCC.

**Patients and Methods:**

From June 2007 to December 2014, 451 patients with potentially resectable thoracic ESCC, clinically staged as T1-4N1M0/T4N0M0, were randomly allocated to NCRT plus surgery (group CRT; n = 224) and surgery alone (group S; n = 227). In group CRT, patients received vinorelbine 25 mg/m^2^ intravenously (IV) on days 1 and 8 and cisplatin 75 mg/m^2^ IV day 1, or 25 mg/m^2^ IV on days 1 to 4 every 3 weeks for two cycles, with a total concurrent radiation dose of 40.0 Gy administered in 20 fractions of 2.0 Gy on 5 days per week. In both groups, patients underwent McKeown or Ivor Lewis esophagectomy. The primary end point was overall survival.

**Results:**

The pathologic complete response rate was 43.2% in group CRT. Compared with group S, group CRT had a higher R0 resection rate (98.4% *v* 91.2%; *P* = .002), a better median overall survival (100.1 months *v* 66.5 months; hazard ratio, 0.71; 95% CI, 0.53 to 0.96; *P* = .025), and a prolonged disease-free survival (100.1 months *v* 41.7 months; hazard ratio, 0.58; 95% CI, 0.43 to 0.78; *P* < .001). Leukopenia (48.9%) and neutropenia (45.7%) were the most common grade 3 or 4 adverse events during chemoradiotherapy. Incidences of postoperative complications were similar between groups, with the exception of arrhythmia (group CRT: 13% *v* group S: 4.0%; *P* = .001). Peritreatment mortality was 2.2% in group CRT versus 0.4% in group S (*P* = .212).

**Conclusion:**

This trial shows that NCRT plus surgery improves survival over surgery alone among patients with locally advanced ESCC, with acceptable and manageable adverse events.

## INTRODUCTION

Esophageal cancer (EC) is the sixth most common cancer worldwide.^1^ China has a high prevalence of EC that accounts for > 50% of the global morbidity and mortality.^2^ More than 90% of patients with EC in China have esophageal squamous cell carcinoma (ESCC). After surgery alone, the prognosis for patients with locally advanced EC remains poor, with a 5-year survival rate of only 25%.^3^

Recent evidence has suggested a survival benefit from neoadjuvant concurrent chemoradiotherapy followed by surgery.^4^ However, the results from randomized controlled trials comparing neoadjuvant chemoradiotherapy (NCRT) followed by surgery with surgery alone have been inconsistent.^5-13^ Moreover, most studies were conducted in Western countries,^5-12^ which have a high prevalence of esophagogastric junction adenocarcinoma. The sample size of ESCC was relatively small in most trials^5-11^; there were usually no more than 80 cases of patients with ESCC receiving NCRT in each study.^5-13^ Whether the results could be applied to East Asian countries such as China where the incidence of ESCC is extremely high remains to be elucidated. A well-designed, large-scale, randomized control trial is needed to evaluate the usefulness of NCRT for ESCC. The current phase III trial enrolled patients with locally advanced ESCC. The primary goal was to compare the survival benefit of NCRT plus surgery versus surgery alone in locally advanced ESCC.

## PATIENTS AND METHODS

### Eligibility

Eligible patients had histologically confirmed, potentially resectable thoracic ESCC clinically staged as T1-4N1M0/T4N0M0 (stage IIB or III) before treatment^14^; were 18 to 70 years of age; had normal hematologic, renal, and hepatic function; and had a Karnofsky performance score of ≥ 90. We excluded patients with a history of other malignancies; those who were unsuitable for surgery because of comorbidities; those for whom reconstruction with stomach conduit was infeasible because of prior gastrectomy; and those unable to sign informed consent because of psychological, family, or social reasons (Appendix, online only).

Approval was obtained from the ethics committee or institutional review board at each center. All included patients provided written informed consent.

### Random Assignment

Patients were randomly assigned in a 1:1 ratio, using a stratified permuted-block method, to receive NCRT followed by surgery (group CRT) or surgery alone (group S) and were stratified according to coordinating centers. Random assignment was generated by computer-generated random assignment lists at the Sun Yat-sen University Cancer Center Clinical Trial Center. The assignments were placed in sealed envelopes, labeled by stratum, which would only be unsealed after patient registration. Permuted-block size was 20. Investigators at each center enrolled participants and assigned them to interventions.

### Pretreatment Workup and Staging

All patients received the following pretreatment examinations and staging: neck, thorax, and abdomen plain and contrast-enhanced computed tomography (CT); esophagogastroduodenoscopy, with ultrasound endoscopy (EUS); and cervical ultrasonography. If indicated, bronchoscopy was performed to exclude tumor infiltration into the trachea or bronchial tree. Positron emission tomography and radionuclide bone imaging were optional.

### Preoperative Chemoradiotherapy

For patients assigned to group CRT, vinorelbine 25 mg/m^2^, intravenous (IV) bolus, days 1 and 8 and cisplatin 75 mg/m^2^, IV within 3 hours, day 1; or 25 mg/m^2^, IV within 2 hours on days 1 to 4 were administered every 3 weeks for two cycles. A total dose of 40.0 Gy was administered in 20 fractions of 2.0 Gy, five fractions per week, starting at the first day of the first cycle of chemotherapy. All patients were radiated by external beam radiation, using the three-dimensional conformal radiation technique. Radiation therapy was delivered with megavoltage equipment with photon energies of 6 to 8 MV. The gross tumor volume was defined by the primary tumor and any enlarged regional lymph nodes, which were determined using all available information (physical examination, endoscopy, EUS, neck-thorax-abdomen CT). The clinical target volume provided a proximal and distal margin of 3 cm and a 0.5- to 1.0-cm radial margin around the gross tumor volume to include the area of subclinical involvement. The planning target volume was defined as an 8-mm margin of the clinical target volume for tumor motion and set-up variations. A detailed description of the methods of chemotherapy and radiotherapy can be found in the protocol. Dose reductions and delays of chemotherapy, and interruptions of radiotherapy, were specified in the protocol (Appendix Table A1, online only). Briefly, full-dose chemotherapy was administered if the absolute neutrophil count was > 1.5 × 10^9^/L and the platelet count was ≥ 75 × 10^9^/L. If not, chemotherapy was delayed for up to 2 weeks until the counts recovered. The second cycle of chemotherapy was discontinued if hematologic toxicity persisted for longer than 2 weeks.

Approximately 4 to 6 weeks after the completion of chemoradiotherapy, patients underwent clinical restaging including physical examination, standard laboratory tests, esophagogastroduodenoscopy with EUS, pulmonary function tests, esophageal barium x-ray, and neck-thorax-abdomen CT.

### Surgery

In group CRT, surgery was scheduled for 4 to 6 weeks after completion of chemoradiotherapy. For patients in group S, surgery was performed as soon as possible after random assignment. McKeown or Ivor Lewis esophagectomy, including two-field lymphadenectomy with total mediastinal lymph node dissection, was performed. The dissection of left and right recurrent laryngeal nerve nodes was mandatory.

### Pathologic Analysis

Reports on pathologic examination should contain the tumor type and extension, proximal and distal resection margins, tumor regression grade (Mandard score), and lymph node status, including the site and the number of nodes with therapy effects. Pathologic complete response (pCR) was defined as no evidence of residual tumor cells in the primary site and resected lymph nodes of the operative specimens.

### Outcomes

The primary end point was overall survival (OS). The time from the date of group assignment to the date of death or the last follow-up was calculated as OS. As for secondary end points, we aimed to compare disease-free survival (DFS), safety profile, rate of R0 resection, and pathologic response. DFS was calculated from the date of R0 resection to the date of disease recurrence or death.

The toxicity of chemotherapy and radiotherapy was evaluated according to the National Cancer Institute Common Terminology Criteria for Adverse Events (version 3.0). Post-treatment follow-up was undertaken in the study centers once every 3 months within the first year and thereafter every 6 months until death or end of study.

### Statistical Analysis

On the basis of our phase II study,^15^ sample size calculations were made assuming a projected median survival of 56 months for patients assigned to group CRT and 39 months for those assigned to group S. With a two-sided type I error of 0.05 and a power of 80%, a randomization ratio of 1:1 between the experimental and control arms, 7 years of accrual, 2 years of follow-up, and two planned interim analyses, and with a 10% dropout rate taken into account, the intended number of randomly assigned patients was 430 (215 per arm). This study was powered to detect a two-sided 5% significance level hazard ratio (HR) of 0.72. The calculations were performed assuming exponential distribution.

All patients randomly assigned to a group (the intention-to-treat population) were included in the primary evaluation of OS. The per-protocol population was defined as all patients who received surgery. We included the per-protocol population in the analysis of postoperative complications. Only the patients who achieved R0 resection were included in the assessment of DFS. We included patients who received concurrent chemoradiotherapy in the analysis for toxicity of chemotherapy and radiotherapy.

**Fig A1.**
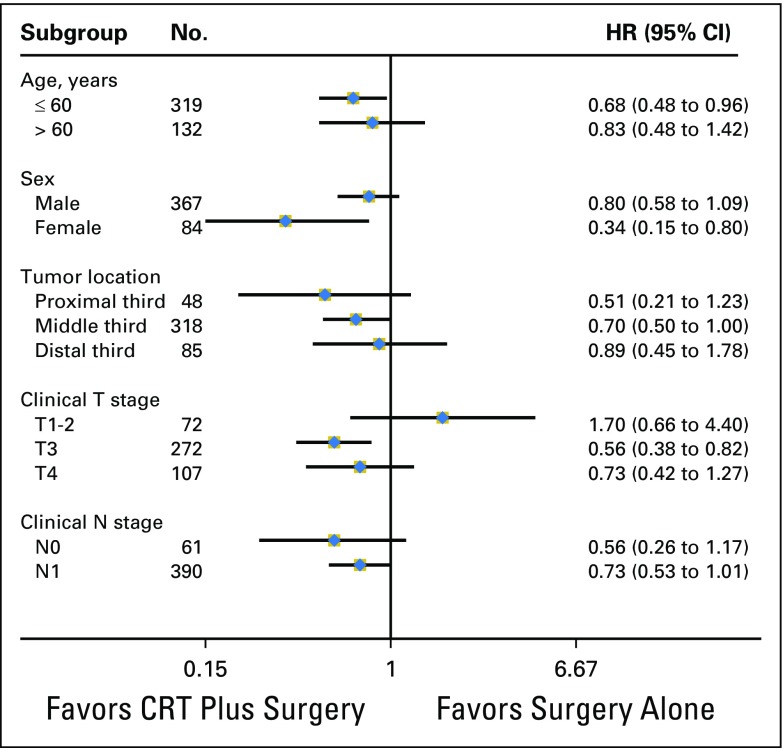
Subgroup analysis. CRT, chemoradiotherapy; HR, hazard ratio.

OS and DFS were calculated using the Kaplan-Meier method and were then compared by the log-rank test. The rate of R0 resection, incidence of complications, and peritreatment mortality were compared with the χ^2^ test or Fisher’s exact test, if indicated. Univariate and multivariate analysis with the Cox proportional hazards model was used to investigate the effect of different factors on survival. Covariates included treatment, age (≤ 60 years *v* > 60 years), sex, tumor location, clinical T stage (T1 to T2 *v* T3 *v* T4) and clinical N stage (Appendix Fig A1, online only). We also used the Cox proportional hazards model to calculate HRs and 95% CIs. We performed two formal interim analyses on Jun 1, 2011, and Dec 31, 2015, after 123 and 451 patients had been enrolled. The significance threshold was defined by the O'Brien-Fleming type boundary 0.000527 in the first interim analysis, 0.014 in the second interim analysis, and 0.045 for the final analysis. The data cutoff for the analysis presented here was December 31, 2016. This trial is registered at ClinicalTrials.gov.

## RESULTS

**Fig 1.**
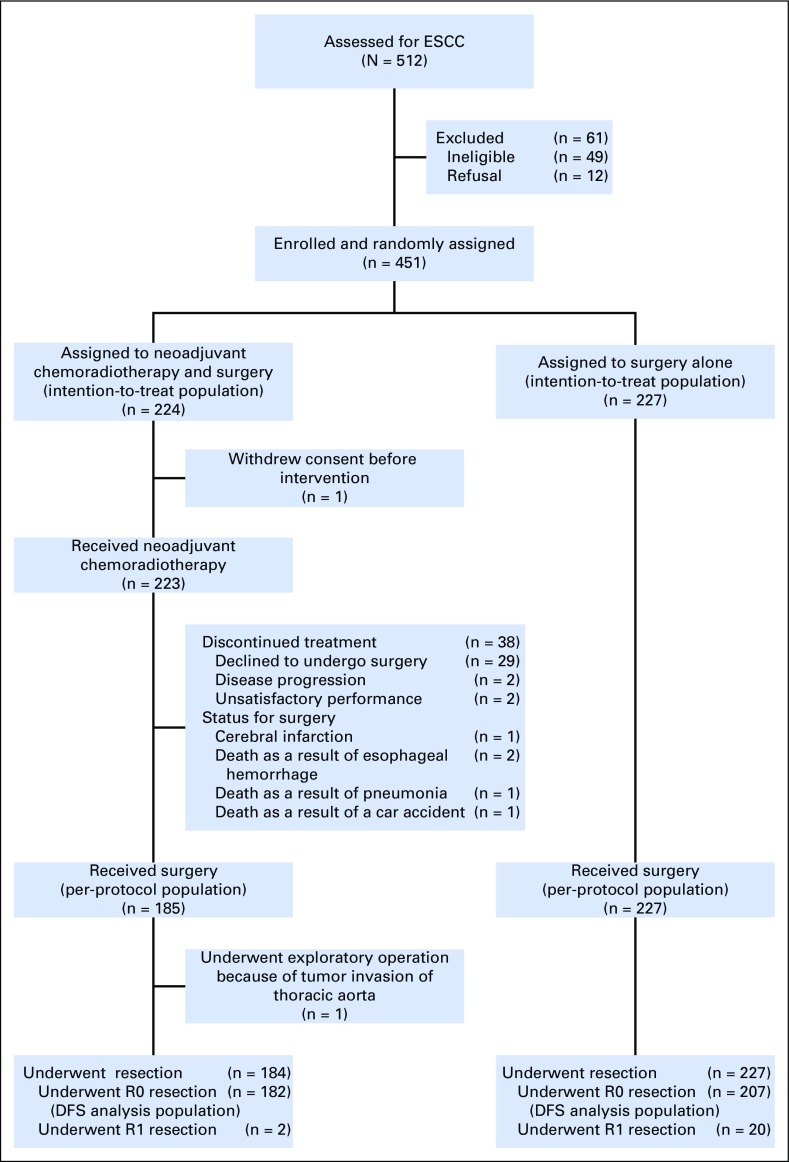
CONSORT diagram. DFS, disease-free survival; ESCC, esophageal squamous cell carcinoma.

From June 2007 to December 2014, 451 patients from eight Chinese centers (Appendix Table A2, online only) were randomly allocated to group CRT (n = 224) or group S (n = 227; Fig 1). The two groups were well balanced at baseline (Table 1).

**Table 1.**
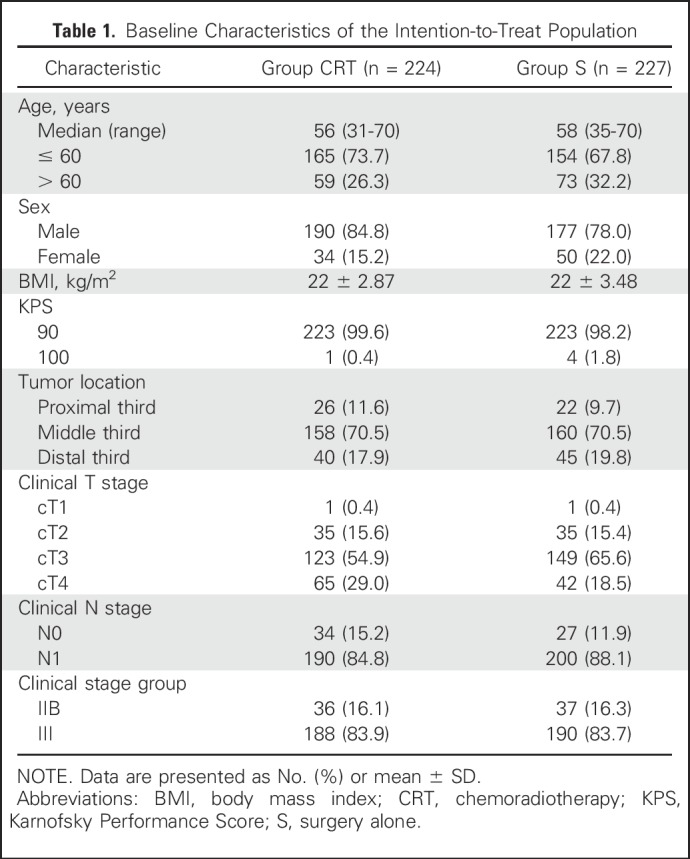
Baseline Characteristics of the Intention-to-Treat Population

### Treatment Compliance

The median NCRT duration was 30 days (interquartile range [IQR], 28-35 days). In group CRT, 185 of 224 patients (82.6%) completed the whole multimodality therapy. Reasons for not undergoing surgery after chemoradiotherapy (38 of 224 [17.0%]) were patient refusal (n = 29), disease progression (n = 2), unsatisfactory performance status for surgery (n = 2), cerebral infarction (n = 1), death as a result of pneumonia (n = 1), death as a result of esophageal hemorrhage (n = 2), and death as a result of a car accident (n = 1). One patient received neither chemoradiotherapy nor surgery because of refusal of all study treatment after random assignment (Fig 1). One hundred and ninety-five patients (87.1%) received two cycles of chemotherapy, whereas 28 (12.5%) received only one cycle (Appendix Tables A3 and A4, online only). Two hundred and twenty-two patients (99.1%) received a total radiation dose of 40.0 Gy. One patient received only 22.0 Gy because of death as a result of pneumonia (Appendix Table A5, online only).

### Safety Profile

Table 2 lists the hematologic and nonhematologic toxicity observed in group CRT. Of 223 patients who received NCRT, 121 patients (54.3%) developed grade 3 or 4 hematologic toxicity, and 16 patients (7.2%) developed grade 3 or 4 nonhematologic toxicity, among which leukopenia and neutropenia were the most common adverse events: 109 patients (48.9%) had grade 3 or 4 leukopenia, and 102 (45.7%) had grade 3 or 4 neutropenia. Postoperative complications did not differ significantly between groups, with the exception of arrhythmia (*P* = .001), which occurred more frequently in group CRT (24 of 185 [13.0%]) than in group S (nine of 227 [4.0%]; Table 3). With regard to peritreatment mortality, 2.2% (five of 224) died in group CRT versus 0.4% (one of 227) in group S (*P* = .212; Appendix Table A6, online only). No death occurred within 30 days after surgery in either group. In group CRT, one of 185 patients (0.5%) died owing to respiratory failure within 90 days postoperatively. In group S, the postoperative 90-day mortality rate was 0.9% (two of 227), which was not significantly different compared with group CRT (*P* = 1.000). One patient died as a result of postoperative respiratory failure, and the other died as a result of out-of-hospital cardiac arrest.

**Table 2.**
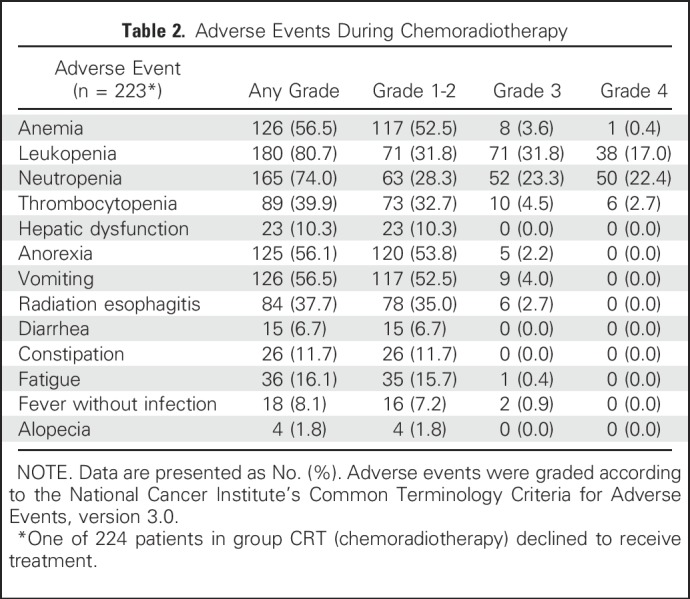
Adverse Events During Chemoradiotherapy

**Table 3.**
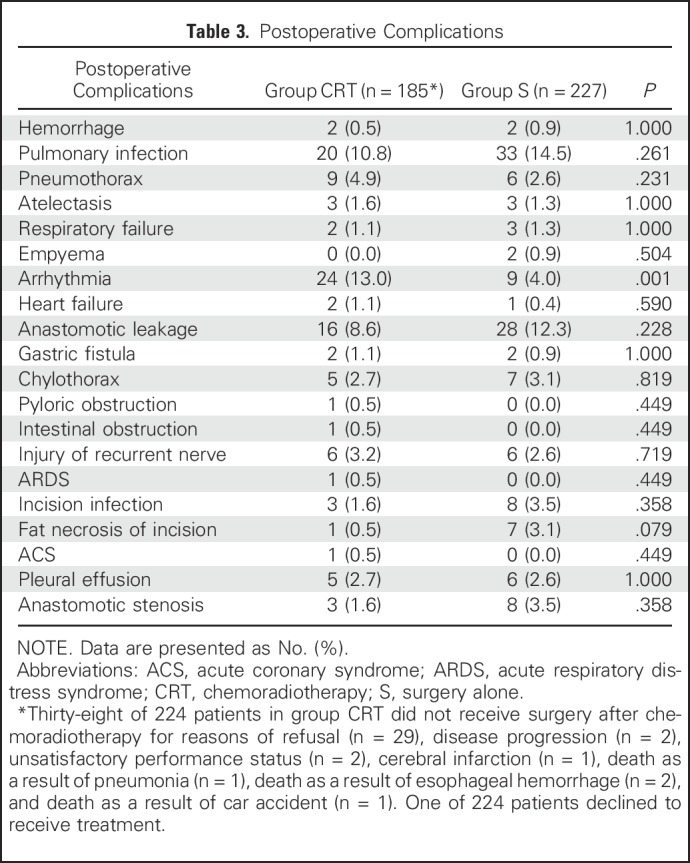
Postoperative Complications

### Surgery

The median interval between the end of NCRT and surgery in group CRT was 1.4 months (IQR, 1.2-1.6 months). The median time between the end of random assignment and surgery was 0.1 month (IQR, 0.03-0.2 month) in group S. Among 185 patients receiving surgery in group CRT, 182 patients (98.4%) underwent R0 resection, compared with 207 of 227 (91.2%) in group S (*P* = .002). In group CRT, one patient underwent an exploratory operation because of tumor invasion of the thoracic aorta. A median of 20 (15 to 27) and 26 (19 to 36) lymph nodes were dissected (*P* < .001), and positive lymph nodes were observed in 61 of 184 patients (33.2%) and 147 of 227 patients (64.8%) in group CRT and group S, respectively (*P* < .001). With respect to the distribution of pathologic stage grouping, patients in group CRT underwent significant downstaging compared with those in group S (*P* < .001): 20 of 185 (10.8%) stage III in group CRT; 142 of 227 (62.6%) in group S (Table 4). A pCR was achieved in 80 of 185 patients (43.2%) after NCRT (Table 4).

**Table 4.**
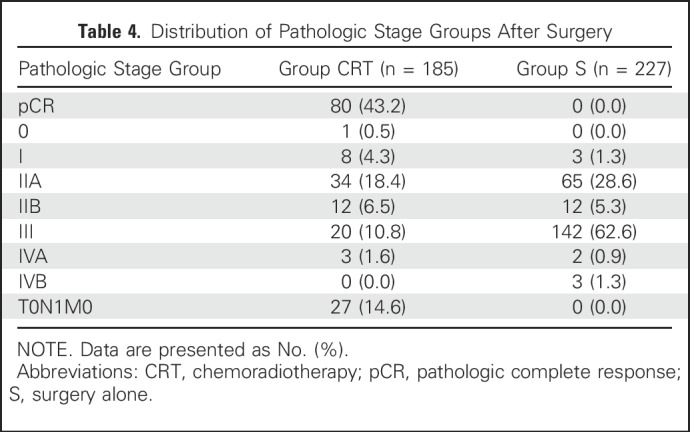
Distribution of Pathologic Stage Groups After Surgery

### Survival

**Fig 2.**
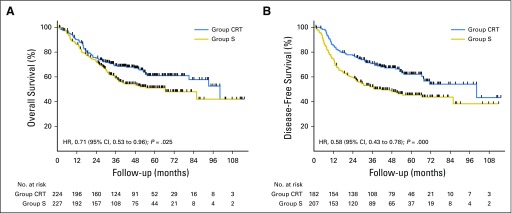
Overall survival and disease-free survival. (A) Overall survival in the intention-to-treat population. (B) Disease-free survival for patients after R0 resection. CRT, chemoradiotherapy; HR, hazard ratio; S, surgery alone.

The median follow-up of the survivors was 41.0 months (IQR, 20.1-59.3 months) in group CRT and 34.6 months (IQR, 17.7-54.2 months) in group S. Kaplan-Meier analysis for OS showed a significant difference between groups. Median OS was 100.1 months (95% CI, 74.6 to 125.6 months) in group CRT versus 66.5 months (95% CI, 39.7 to 93.3 months) in group S (HR, 0.71; 95% CI, 0.53 to 0.96; *P* = .025). The OS rates in group CRT and group S were 90.0% (95% CI, 85.2% to 93.3%) and 86.2% (95% CI, 80.9% to 90.1%) at 1 year; 75.1% (95% CI, 68.8% to 80.4%) and 72.5% (95% CI, 66.1% to 77.9%) at 2 years; 69.1% (95% CI, 62.4% to 74.8%) and 58.9% (95% CI, 52.0% to 65.3%) at 3 years, respectively. Among the 389 patients undergoing R0 resection, the median DFS was 100.1 months (95% CI, 49.7 to 150.6 months) in group CRT, compared with 41.7 months (95% CI, 19.0 to 64.4 months) in group S (HR, 0.58; 95% CI, 0.43 to 0.78; *P* < .001; Fig 2). Multivariate analysis that was based on the intention-to-treat population showed that NCRT plus surgery and lower T stage independently predicted better survival (Table 5).

**Table 5.**
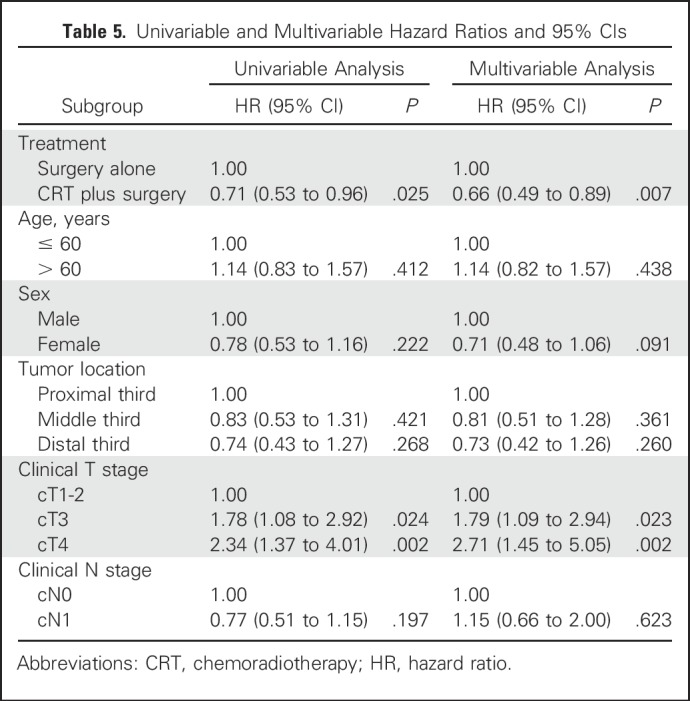
Univariable and Multivariable Hazard Ratios and 95% CIs

## DISCUSSION

This phase III clinical trial demonstrates that, compared with surgery alone, NCRT followed by surgery significantly increased OS as well as DFS in patients with locally advanced ESCC. A preoperative chemoradiotherapy regimen that was based on vinorelbine and cisplatin was manageable and had a favorable safety profile. Compared with those treated with surgery alone, patients treated with NCRT followed by surgery had a similar postoperative complication rate and peritreatment mortality, whereas the risk of death during follow-up was 29% lower (HR, 0.71; 95% CI, 0.53 to 0.96).

This study followed the same NCRT protocol as that used in our previous phase II trial.^15^ In that study, R0 resection was achieved in 98% of patients. In the phase II trial, IV administration of cisplatin was used at 75 mg/m^2^ on day 1.^15^ This dose of cisplatin required high-volume hydration over 5 hours to prevent renal dysfunction, which can be inconvenient for outpatient chemotherapy. Therefore, the current phase III trial also allowed for the IV administration of cisplatin at 25 mg/m^2^ on days 1 to 4, which did not require hydration and could be used in an outpatient department. In the study, the clinicians in charge selected one of the protocols, and OS was comparable between the two protocols (Appendix Tables A7-A11, online only).

This study was designed to detect a difference of 17 months in median survival in favor of NCRT, as compared with surgery alone (56 months *v* 39 months). The final results showed that OS in both groups was further improved and the outcome was better than previously reported.^5-13^ In addition, the therapeutic efficacy of surgery alone was also better than that reported in the previous study.^5-13^ A possible explanation is that this trial implemented total mediastinal lymph node dissection, especially recurrent laryngeal nerve node dissection, which was not required in previous trials.^5-13^ For patients with ESCC, the metastatic rate of recurrent laryngeal nerve lymph nodes ranges from 20% to 40%.^16,17^ Furthermore, the perioperative mortality rate was lower than in previous trials.^5-13^ This may be attributed to the fact that centers in East Asian countries have developed more extensive clinical experience in the treatment of EC because of the higher incidence and prevalence of EC in East Asia and that operations are performed in high-volume centers. Of note, the perioperative mortality rate in other studies from East Asia was also no higher than 2%.^18-20^ In this study, the OS (67.2% for 3-year OS) in group CRT is in line with the prognosis (68.3%) of the squamous cell carcinoma subgroup receiving NCRT in the ChemoRadiotherapy for Esophageal Cancer Followed by Surgery Study (CROSS).^11^ Taken together, these results suggest that the difference in OS should not be ascribed to the poor outcomes in group S, but can be attributed to effective NCRT, followed by surgery.

There have been conflicting results from previous studies comparing the efficacy of NCRT with surgery alone in patients with EC,^5-13^ especially in those with ESCC. In many countries, both NCRT plus surgery and surgery alone are standard treatments for patients with locally advanced ESCC.^21-23^ The CROSS phase III trial indicated that NCRT followed by surgery significantly improved OS in patients with esophageal or esophagogastric junction cancer when compared with surgery alone.^11^ However, the benefit of NCRT for ESCC was questioned, because it was observed in a relatively small subset of patients (84 patients [23% of the total recruited number]). In addition, the low R0 resection rate in group S (69%) and the low 5-year survival rate for patients with ESCC treated with surgery alone (28%) have brought into question the robustness of the results in this subgroup of patients.^11^ In the same period, although the majority of patients in the FFCD 9901 trial had ESCC (137 patients [70% of the total patients recruited]), the result showed that the OS was not different between the NCRT plus surgery group and group S. Compared with the CROSS trial, this study recruited 451 patients with ESCC, and > 90% of patients received R0 resection in group S. Thus, the significant difference in survival was not ascribed to a low rate of R0 resections. Patients with locally advanced EC were recruited for this study, which was different from the study design of FFCD 9901, in which patients with lower stages were enrolled. Moreover, 82.6% of patients completed the full neoadjuvant treatment protocol with a peritreatment mortality rate of 2.2%. Thus, safety and treatment compliance were favorable.

There are several factors that contributed to the significant survival benefits of NCRT observed in this study. First, shrinkage of the primary tumor and lymph node metastases after chemoradiotherapy significantly increased the R0 resection rate, which is an independent prognostic factor.^24,25^ Second, 43.2% of patients achieved pCR after NCRT, which is in line with previous reports,^11,12^ and these patients benefited most from neoadjuvant therapy.^26^ Third, patients with locally advanced EC were recruited for this study. These patients have a high tumor burden and are more likely to have micrometastasis, and thus may receive greater oncologic benefit from NCRT. Fourth, compared with surgery alone, the preoperative chemoradiotherapy of this study did not significantly increase the postoperative morbidity and mortality. Therefore, the survival benefit from NCRT was not counteracted by chemoradiation-induced adverse events.

This trial has several limitations. Patients with poorer performance status and older patients were not recruited, and the applicability of this combined therapy to these patients requires additional study. The study was conducted in China, which has a high prevalence of ESCC, and whether these results are applicable in Western countries with a high prevalence of esophagogastric junction adenocarcinoma warrants additional investigation.

In conclusion, NCRT according to the NEOCRTEC5010 regimen is safe and significantly prolongs OS and DFS in patients with locally advanced ESCC, compared with surgery alone. We believe that this study’s findings are important for policy revising and decision making when choosing the treatment for patients with potentially resectable, locally advanced ESCC.

## Figures and Tables

**Table A1.**
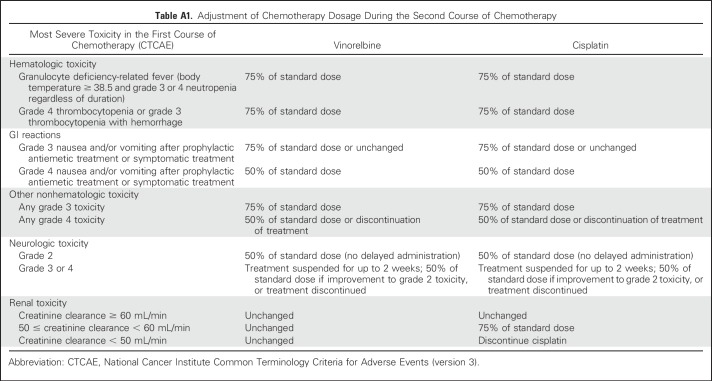
Adjustment of Chemotherapy Dosage During the Second Course of Chemotherapy

**Table A2.**
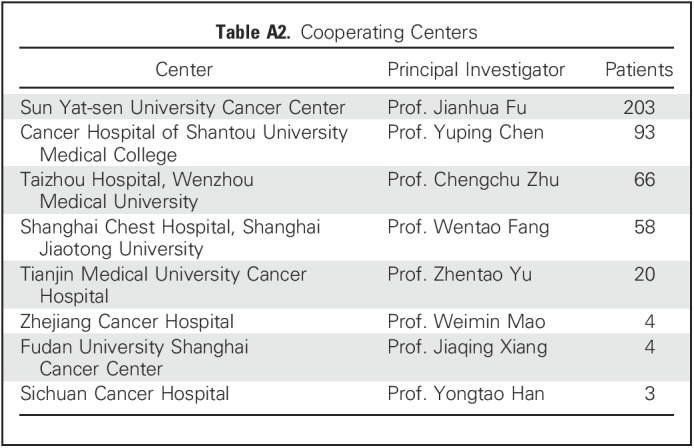
Cooperating Centers

**Table A3.**
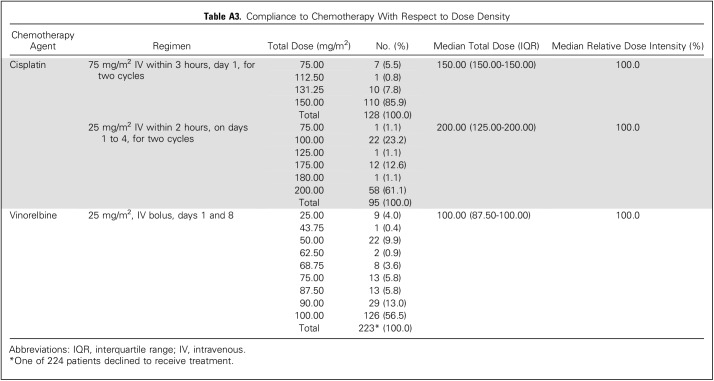
Compliance to Chemotherapy With Respect to Dose Density

**Table A4.**
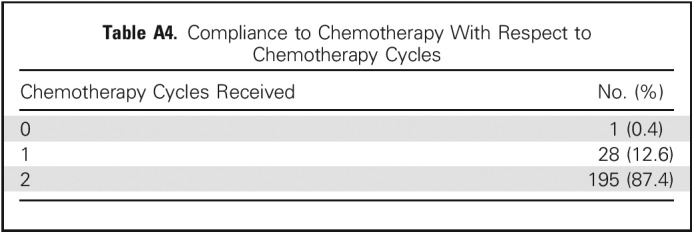
Compliance to Chemotherapy With Respect to Chemotherapy Cycles

**Table A5.**
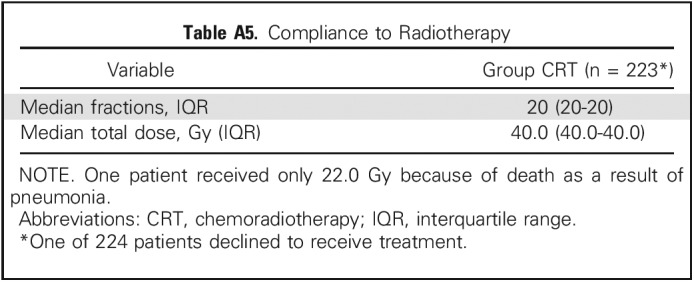
Compliance to Radiotherapy

**Table A6.**
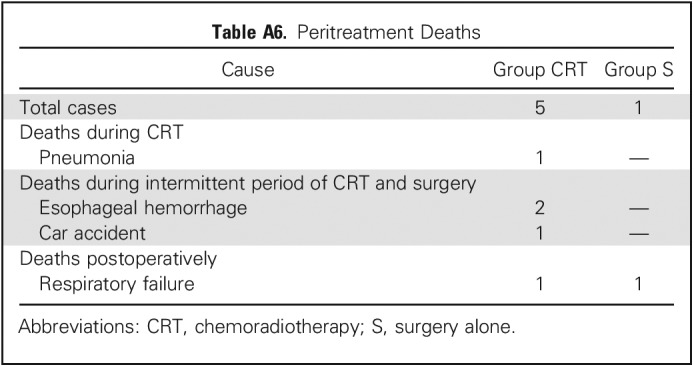
Peritreatment Deaths

**Table A7.**
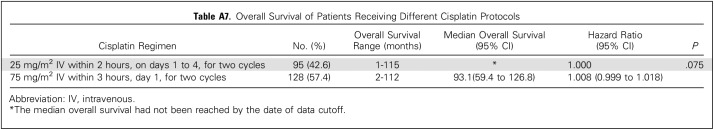
Overall Survival of Patients Receiving Different Cisplatin Protocols

**Table A8.**
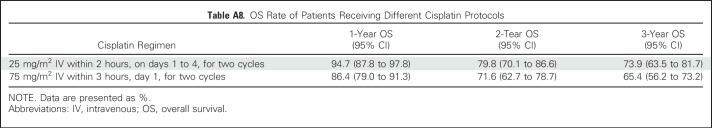
OS Rate of Patients Receiving Different Cisplatin Protocols

**Table A9.**
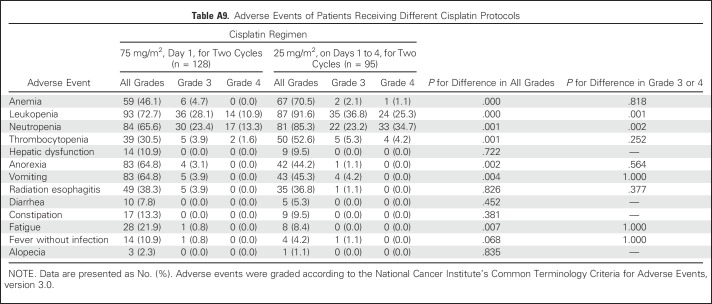
Adverse Events of Patients Receiving Different Cisplatin Protocols

**Table A10.**
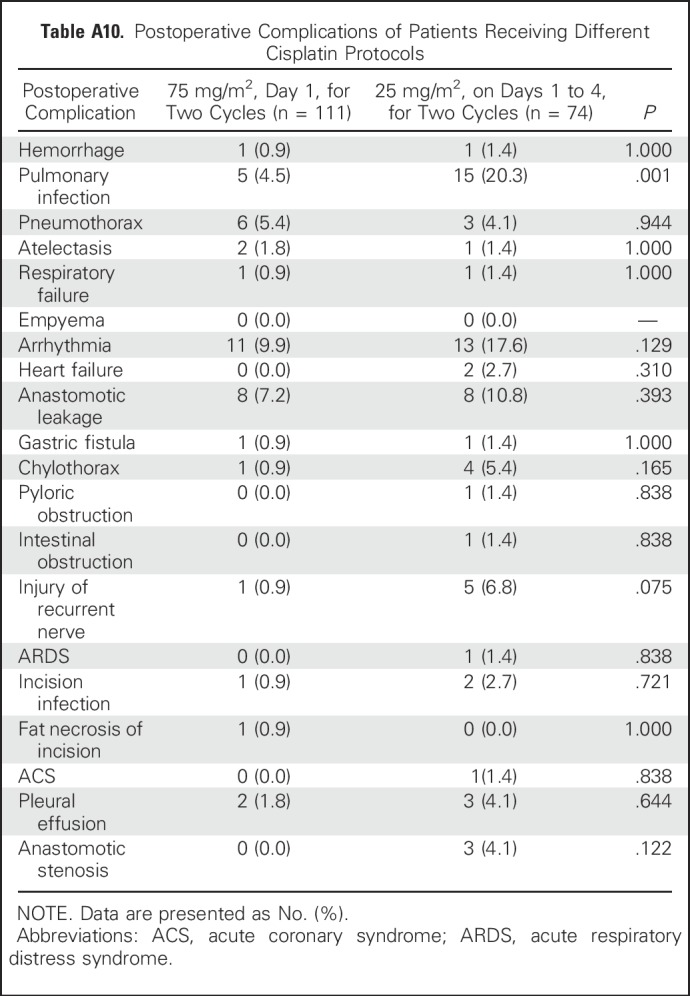
Postoperative Complications of Patients Receiving Different Cisplatin Protocols

**Table A11.**
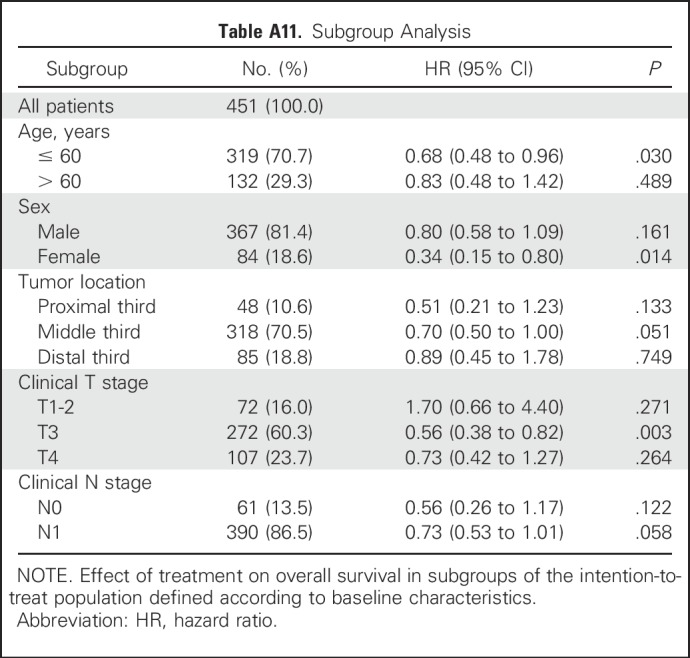
Subgroup Analysis

## Appendix

### Patient Selection

#### Inclusion Criteria.

Diagnosed with potentially resectable stage IIb or III thoracic esophageal squamous cell carcinoma (according to the American Joint Committee on Cancer [6th edition])
No previous treatment
At least 6 months of expected survival
Between 18 and 70 years of age
Adequate marrow: WBC ≥ 4.0 × 10^9^/L; neutrophil ≥ 1.5 × 10^9^/L; platelet ≥ 100.0 × 10^9^/L; hemoglobin ≥ 90 g/L
Normal liver and kidney function
Satisfactory performance status: Karnofsky performance score ≥ 90
From whom informed consent will be obtained before the study

#### Exclusion Criteria.

Prior treatment to primary tumor or nodes
Allergic history or suspicious allergy to chemotherapy agents such as diamminedichloroplatinum (cisplatin) and vinorelbine
History of or concomitant hemorrhagic diseases
For whom surgery is not allowed because of other uncontrollable diseases
Pregnant or lactating
Incapable of signing informed consent because of psychological, family, or social reasons
For whom reconstructions with stomach as the conduit are infeasible because of prior surgery
Peripheral neuropathy and the Common Terminology Criteria for Adverse Events (version 3.0) grade is ≥ 2
Prior malignancies except for adequately treated basal cell or squamous cell skin cancer, in situ cervical cancer

#### Criteria for Removal From Protocol Treatment.

Distant metastasis present during treatment
Intercurrent disease, which would affect assessments of clinical status to a significant degree, require discontinuation of drug, or both
Unacceptable toxicity
Patient becomes intolerant of surgery after preoperative chemoradiotherapy
Patient may withdraw from the study at any time for any reason

### Investigators and Research Staff

Yonghong Hu, Qiaoqiao Li, Mian Xi, Liru He, Bo Qiu, Shiliang Liu, Xiaodong Li, Kongjia Luo, Wenfeng Ye, Jing Wen, Xuan Xie, Fu Yang, Ruiqi Wang, Feixiang Wang, Jiyang Chen, Junying Chen, Yihuai Hu, Shihua Yao, Peng Tang, Zhao Ma, Longlong Shao, and Lin Peng.

## Independent Data Monitoring Committee

Tiehua Rong, Qing Liu, and Yuhong Li.

## Other Advisors

Jang Ming Lee, Linquan Tang, and Shaodong Hong.
